# Effect of a functional food (vegetable soup) on blood rheology in patients with polycythemia

**Published:** 2018

**Authors:** Hamideh Naghedi-Baghdar, Mohsen Nematy, Mohammad-Mahdi Kooshyar, Ali Taghipour, Sayyed Abolghasem Sajadi Tabassi, Sadegh Shokri, Roghayeh Javan, Seyed-Mohammad Nazari

**Affiliations:** 1 *Student Research Committee, School of Persian and Complementary Medicine, Mashhad University of Medical Sciences, Mashhad, Iran*; 2 *Biochemistry and Nutrition Research Center, Faculty of Medicine, Mashhad University of Medical Sciences, Mashhad, Iran*; 3 *Department of Internal Medicine, Faculty of Medicine, Mashhad University of Medical Sciences, Mashhad, Iran*; 4 *Department of Biostatistics and Health Sciences Research Center, School of Health, Mashhad University of Medical Sciences, Mashhad, Iran*; 5 *Pharmacological Research Center of Medicinal Plants, Faculty of Medicine, Mashhad University of Medical Sciences, Mashhad, Iran*; 6 *Department of Persian and Complementary Medicine, School of Persian and Complementary Medicine, Mashhad University of Medical Sciences, Mashhad, Iran*; 7 *Traditional and Complementary Medicine Research Center, Sabzevar University of Medical Sciences, Sabzevar, Iran *

**Keywords:** Blood rheology, Blood viscosity, Polycythemia, Functional food, Diet

## Abstract

**Objective:**

Key hemorheological variables are associated with several life-threatening diseases including cardio-cerebro-vascular diseases. A diet can influence the blood rheological variables. To compare the effectiveness of a vegetable soup on blood viscosity (BV), hematocrit (Hct), plasma fibrinogen, lipid profile, fasting blood sugar (FBS), and blood osmolarity in patients with polycythemia in comparison to a control group.

**Materials and Methods:**

This randomized controlled trial study was conducted at Isar health clinics in Mashhad, Iran, during a 7-month period. Forty male participants (35 to 60 years old) with polycythemia, but without underlying diseases, were included. They randomly assigned to two groups and either received diet/phlebotomy or phlebotomy alone, for 6 weeks. The data were analyzed by SPSS version 16 using parametric tests.

**Results:**

A significant reduction in BV at 30s (p≤0.001), BV at 40s (p≤0.001), BV at 50s (p≤0.001), Hct (p≤0.001), plasma fibrinogen (p≤0.001), total cholesterol (p<0.01), LDL-cholesterol (p<0.01), VLDL-cholesterol (p≤0.001), HDL-cholesterol (p≤0.01), osmolarity (p≤0.001), and FBS (p≤0.001) was observed in diet recipients. Following the intervention, there was a significant decrease in triglyceride (intervention group, p<0.05; control group, p<0.05), in both groups.

**Conclusion:**

This trial showed that the plant–based food used in this study could improve blood rheology.

## Introduction

Hemorheology is the study of the blood flow, its deformation behavior and its formed elements (Hamlin and Benedik, 2014 ▶). Key rheological variables include blood viscosity as a dynamic property of the blood and hematocrit, plasma proteins, lipid profiles, glucose, creatinine and osmolality, as static properties of the blood (Holsworth Jr et al., 2014 ▶). Hematocrit (Hct) and plasma fibrinogen are major determinant of plasma and blood viscosity (Peters et al., 2016 ▶). Scientific evidence has shown that the rheological properties of the blood are associated with pathogenesis, development, and prognosis of several life-threatening diseases. Some of the most important of these conditions are cardiovascular disorders, myocardial infarction, transient ischemic attack, arterial hypertension, diabetes mellitus, haemorrhagic shock, renal diseases, dyslipidemia, and sickle cell anemia (Hitsumoto, 2012 ▶; Sousa et al., 2016 ▶). Two therapeutic procedures are available for decreasing blood viscosity: direct and indirect approaches. The direct methods include plasma exchange, phlebotomy, and rheopheresis and the indirect therapy acts through regulating the factors that influence blood viscosity, such as erythrocytes, platelets, and endothelial cells (Chen et al., 2012 ▶; Mandal, 2016 ▶).

Diet is one of the defined factors for low-risk lifestyle (Larsson et al., 2014 ▶). Functional foods which are defined as food products with special constituents and beneficial physiological effects, play a crucial role in prevention and treatment of chronic diseases (Martirosyan and Singh, 2015 ▶). The relationship between some nutritional–related diseases such as hypertriglyceridemia, hypoalbuminemic disorders, and diabetes mellitus, and blood and plasma viscosity has been demonstrated in several studies (Carallo et al., 2013 ▶; Sloop et al., 2015 ▶). New scientific findings concerning coronary artery disease show that the risk of this disease decreases with increased consumption of plant–based foods and decreased consumption of animal–based foods (Tuso et al., 2013 ▶). Vegetable soup as a functional food is rich in bioactive components such as antioxidants, dietary fibers, vitamins, minerals, essential fatty acids and oligosaccharides. Ingredients that make vegetable soup contribute to prevent many chronic health problems such as cardiovascular and inflammatory diseases (Lobo et al., 2010 ▶). 

In this experiment, a randomized clinical trial study was carried out to assess the hemorheological effect of a functional food which was prepared based on Persian medicine and contained onion, celery, lettuce, carrot, barley, parsley, coriander, dill, extra virgin olive oil, lime juice, cinnamon, turmeric, salt and black pepper, on whole blood viscosity (WBV), Hct, plasma fibrinogen, lipid profile, FBS, and blood osmolarity in patients with polycythemia.

## Materials and Methods

**Trial design and participants**

This randomized clinical trial was carried out during a 7-month period (year 2016) at Isar health clinics affiliated to Imam Reza hospital, Mashhad University of Medical Sciences, Mashhad, Iran. The study was registered in the Iranian Registry of Clinical Trials (IRCT2015111024993N1). The study population comprised of male patients, who referred to the hematology clinic of Isar health clinics, and diagnosed with polycythemia without underlying diseases. Women were not recruited because premenopausal women compared with men have significantly lower WBV, Hct and red blood cell aggregation and rigidity, due to regular menstrual blood loss of approximately 50-100 mL per cycle (Holsworth et al., 2014 ▶). 

**Selection criteria**

Male patients with polycythemia (Hct ≥50) without underlying diseases such as renal, respiratory, cardiovascular and liver diseases, malignancies, hyperlipidemia and diabetes mellitus, aging 35 to 60 years old, not taking any medication during the last two weeks before entering the study, were enrolled into this trial. The exclusion criteria were food allergy; cigarette, tobacco, and narcotics smoking; underlying diseases mentioned above; hospitalization; use of drugs that affect blood rheology such as aspirin; bloodletting or leech therapy during the study; lack of consent to continue the project and possible side effects. 

**Interventions**

A diet regimen for 6 weeks and phlebotomy at baseline considered for intervention group. This diet was served as dinner and no other food was served within the next two hours. One's daily serving consumption was determined based on BMI (kg/m^2^): 300 grams daily for subjects with a BMI<20, 350 grams daily for those with a 20<BMI<30, and 400 g daily for those with a BMI>30. Diet components were purchased from city stores. Food ingredients were weighed, and the food was prepared according to the recipe. The control group received only routine treatment (phlebotomy) at the beginning of the study. A blood donation of 400 mL was done by both groups. All subjects were instructed to maintain their usual diet but they were not allowed to consume Olivier salad, pasta, boiled potato, fast foods, fried foods, and canned foods during the last two weeks before entering the study and continued the same regimen during the study period (6 weeks).

**Diet components**

Diet components included onion (40 g, 22.1%), celery, lettuce and carrots (25 g each, 13.8%) barley (20 g, 11.1%) parsley, coriander and dill (10 g each, 5.5%), extra virgin olive oil and lime juice (7 g each, 3.9%) cinnamon, turmeric and salt (0.5 g each, 0.3%) and black pepper (0.3 g, 0.2%) (Aghili MH, 2009). A complete serving (181 g) typically had 132 kcal, and included fat (69 kcal), carbohydrates (44 kcal), protein (10 kcal), and fibers (9 kcal). Analysis of selected nutrients of raw ingredients based on Dietplan software; version 7.00 (30 day trial) is shown in Table 1. 

**Table 1:** Analysis of selected nutrients.

**Nutrient**	**Unit**	**Total**	**Per 100g**
**Water g 151.4 83.7**	g	151.4	83.7
**Total Nitrogen g 0. **	g	0.42	0.23
**Protein**	g	2.6	1.4
**Fat**	g	7.7	4.3
**Carbohydrate (mse)**	g	11.7 +	6.5
**Energy (kcal)**	kcal	123 +	68
**Starch (mse)**	g	5.6 +	3.1
**Total Sugars (mse)**	g	5.2 +	2.9
**Glucose**	g	2.0 +	1.1
**Fructose**	g	1.6 +	0.9
**Sucrose (mse)**	g	1.6 +	0.9
**Non-starch polysaccharides**	g	2.5 +	1.4
**Total dietary fiber (AOAC method)**	g	4.3 +	2.4
**Saturated fatty acids**	g	1.1 m	0.6
**Mono-unsaturated fatty acids**	g	5.1 m	2.8
**Poly-unsaturated fatty acids**	g	0.7 m	0.4
**Total Trans fatty acids**	g	----	----
**Cholesterol**	mg	----	----
**Sodium (Na)**	mg	232	128
**Potassium (K)**	mg	472	261
**Calcium (Ca)**	mg	110	61
**Magnesium (Mg)**	mg	25	14
**Phosphorus (P)**	mg	62	35
**Iron (Fe)**	mg	2.12	1.17
**Zinc (Zn)**	mg	0.68	0.38
**Total Folate**	µg	54 +	30
**Carotene**	µg	3379	1869
**Vitamin C **	mg	36	20
**Vitamin D**	µg	Nil	Nil
**Vitamin E**	mg	1.46 +	0.81
**Vitamin B12 **	µg	Nil	Nil

**Recipe**

First, the barley was cooked with a liter of water at gentle heat for half an hour. Then, carrot and celery were added, and after 15 min, lettuce, parsley, dill, coriander, pepper, turmeric, cinnamon, salt, and olive oil were added and cooked for 15 min. Then, the flame was turned off and after a while lemon juice was added. 

**Assessment of dietary intake**

Each participant completed a questionnaire during the study. The subjects were instructed to record the number of days and the amount they consumed this food. Participants were also asked to announce if their routine diet had changed (e.g. they did not eat lunch one day). Additionally, the subjects filled a questionnaire concerning the side effects such as nausea, vomiting, diarrhea and food allergy. 

**Outcomes**

The primary outcomes were changes in BV, Hct, and plasma fibrinogen before and after intervention. The blood viscosity was measured at three times in seconds 30, 40, and 50. The secondary outcomes were measurement of lipid profile, FBS, blood osmolarity before and after the intervention, and adverse effects during the course of treatment.

**Measuring methods**

Blood samples were collected from the antecubital vein in a sitting position after 12-hr fasting. Whole blood viscosity was determined within 1 hr after sample collection. WBV was measured by the R/S plus Coaxial Cylinder Rheometer (AMETEK Brookfield, USA) using CC-14 cup and bob geometry. Other parameters were assessed within 2 hr after sample collection. Hematocrit was measured by flow-cytometry using an automated hematology analyzer, Celltac ES (Nihon Kohden Corporation- Tokyo- Japan). Fibrinogen was evaluated with a coagulometery method using the Stago coagulation analyzer, STA R MAX, (Stago- Australia / New Zealand). Lipid profile, FBS and urea were measured using a turbidometry method by an autoanalyzer, Alpha Classic- 6 model, (Tajhizat Sanjesh Co., Ltd- Isfahan- Iran). Blood osmolarity was calculated based on the following formula:

[Serum Na^+^ (mmol/L)×2] + [glocose (mg/dL)÷18] + [urea (mg/dL)÷2.8 ]

Sodium was measured by Caretium, an automatic electrolyte analyzer, (Caretium Medical Instruments Co, Limited- China).

**Sample size**

According to the previous studies, the planned sample size was 20 participants in each group (Kalus et al., 2000; Nozawa et al., 2007; Sofi et al., 2010). In all calculations, the H0-hypothesis was disapproved if the error probability (p) was below 0.05.

**Randomization**

This was a randomized clinical trial study. The patients were randomly divided into two equal groups according to the random number table generated by computer. It was explained to patients that they were allocated in two different treatment groups, but they did not know about the main intervention. Allocation concealment was kept until the end of the study. Both assessor and the statistician were aware of the allocations. After being randomly allocated to the groups, preliminary tests of participants were assessed by a unit laboratory. 

**Statistical analysis**

The collected data were analyzed by SPSS software version 16.0 (SPSS, Inc., Chicago, IL, USA). All values are expressed as mean±standard deviation (SD). Normality analysis were done using Kolmogorov-Smirnov test. After ensuring normal distribution of variables, parametric tests were used. In order to achieve the goals of inferential statistics analysis, paired t-test and independent t-test were used. Independent t-test was used to analyze differences between changes observed between intervention and control group. Paired t-test was used to compare the mean values of the two groups before and after intervention.

## Results

**Figure 1:**
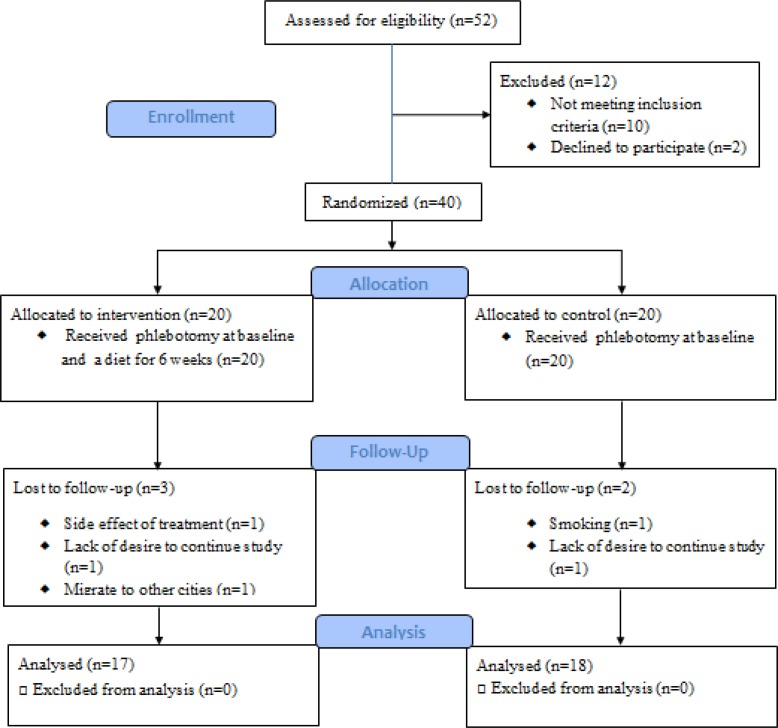
CONSORT flow diagram of the trial.

The study CONSORT flowchart is shown in Figure 1. Among the 52 referred patients to the Isar health clinics, 40 patients were enrolled based on the inclusion criteria. They were randomized into two 20-patient groups of intervention (group 1) and control (group 2). Seventeen participants (85%) in group 1 (49.18±6.96 years old; BMI= 25.76±2.05); and eighteen participants (90%) in group 2 (45.61±7.98 years old; BMI= 25.73±2.18) completed the study. The patients’ average age and BMI distribution did not show significant differences when comparing the two groups (p=0.17 and p=0.97, respectively). 

Changes in variables were separately investigated before and after the treatment in each group and compared with the other group.

**Whole blood viscosity, hematocrit and plasma fibrinogen**

The influence of vegetable soup on blood viscosity, Hct and plasma fibrinogen is shown in Table 2.

A significant influence of vegetable soup on blood viscosity at seconds 30 and 40 was found only in intervention group (p<0.001). There was a significant decrease in blood viscosity at second 50 (group 1, p<0.001; group 2, p=0.02), Hct (group 1, p<0.001; group 2, p<0.001), and plasma fibrinogen (group 1, p<0.001; group 2, p=0.001), before and after the intervention in both groups.

Comparison of variables values before and after the study showed a significant improvement in blood viscosity at seconds 30 (p<0.001), 40 (p<0.001) and 50 (p<0.001), Hct (p<0.001), and plasma fibrinogen (p<0.001) in intervention group compared to the other group.

**Lipid profile, Osmolarity, and FBS**

The influence of vegetable soup on total cholesterol (TC), LDL-cholesterol, VLDL-cholesterol, HDL-cholesterol, triglyceride (TG), osmolarity, and FBS is shown in Table 3.

After ingestion of vegetable soup, there was a statistically significant decrease in only intervention group in TC (p<0.001), LDL-cholesterol (p<0.001), VLDL-cholesterol (p<0.001), HDL-cholesterol (p<0.01), osmolarity (p<0.001), and FBS (p<0.001), whereas no significant changes were observed in the control group. There was a significant decrease in TG (group 1, p<0.05; group 2, p<0.05), when comparing before and after the intervention in both groups.

The difference between before and after the study values demonstrated a significant improvement in TC (p<0.01), LDL-cholesterol (p=0.01), VLDL-cholesterol (p<0.001), osmolarity (p=0.001), and FBS (p=0.001) in intervention group, whereas no significant difference was observed in HDL-cholesterol.

**Adverse effects**

Nausea was seen in one participant of intervention group who was excluded from the study.

**Table 2:** Effect of dietary interventions on blood viscosity, hematocrit and plasma fibrinogen.

**Variable**	**Control group**			**Intervention group**		
	**Before**	**After**	**Change**	**Before**	**After**	**Change**
**WBV at 30s, mPa.S **	6.350.58	6.280.61	0.060.5	6.540.51	5.770.3 ***	0.760.45^+++^
**WBV at 40s, mPa.S **	4.600.45	4.480.30	0.120.36	4.590.30	4.080.12 ***	0.510.25 ^+++^
**WBV at 50s, mPa.S **	3.620.24	3.480.18*	0.140.22	3.650.23	3.120.14 ***	0.540.15 ^+++^
**Hematocrit, % **	50.640.52	45.520.61***	5.110.85	50.870.62	44.630.61 ***	6.230.81 ^+++^
**FG, mg/dL **	296.8367.77	281.3353.68 ***	15.517.04	312.1263.86	227.037.85 ***	85.1252.45 ^+++^

WBV: Whole Blood Viscosity, FG: fibrinogen

* p<0.05,

** p<0.01, and

*** p<0.001show significant differences as compared to “before intervention value”.

+ p<0.05,

++ p<0.01, and

+++ p<0.001show significant difference changes as compared to control group.

**Table 3:** Effect of dietary interventions on lipid profile, osmolarity, and FBS.

**Variable**	**Control group**			**Intervention group**		
	**Before**	**After**	**Change**	**Before**	**After**	**Change**
**TC, mg/dL **	199.2834.27	194.6134.71	4.6611.6	194.7636.48	178.2430.76***	16.5310.31^++^
**LDL-C, mg/dL **	112.1717.5	108.1118.46	4.0610.4	112.5925.87	98.8220.14 ***	13.7611.38 ^++^
**VLDL-C, mg/dL **	46.667.12	45.358.46	1.313.92	41.8212.1	33.75 12.1 ***	8.15.9^+++^
**HDL-C, mg/dL **	37.283.29	37.943.32	0.663.07	37.006.16	40.125.82**	3.124.25
**TG, mg/dL**	177.2229.43	172.0631.52*	5.1610.28	151.7170.41	130.1872.26*	21.5333.33
**OS, ** **mOsm/L**	294.144.83	294.685.49	0.555.23	296.533.99	291.154.77 ***	5.383.77 ^+++^
**FBS, mg/dL **	94.8911.73	92.9414.20	1.948.77	102.6514.45	89.1210.34 ***	13.539.64 ^+++^

TC: Total Cholesterol, LDL-C: Low-Density Lipoprotein-Cholesterol, VLDL: Very Low-Density Lipoprotein-Cholesterol, HDL: High-Density Li

poprotein-Cholesterol, TG: Triglyceride, OS: Osmolarity, FBS: Fasting Blood Sugar.

* p<0.05,

** p<0.01, and

*** p<0.001 show significant differences as compared to “before intervention value”.

++ p<0.01, and

+++ p<0.001show significant difference changes as compared to control group.

## Discussion

The present study examined the effects of a vegetable soup on several human health parameters. Main findings were significant reductions in WBV, Hct, plasma fibrinogen, TC, LDL-cholesterol, VLDL-cholesterol, HDL-cholesterol, osmolarity, and FBS in intervention group. Also, there was a significant decrease in TG in both groups. 

Based on our literature search, some studies have examined the effectiveness of the diet on blood rheology, but no studies conducted to evaluate a plant-based food on patients with polycythemia, were found. A randomized double-blind placebo-controlled study was performed by Yoshizu Nozawa et al. (2007) in twenty-four healthy adult subjects. The subjects ingested the dried-bonito broth (DBB) or placebo for four weeks, and blood fluidity and oxidative stress were measured before and after the study period. DBB ingestion significantly ameliorated the blood fluidity (Nozawa et al., 2007 ▶). Kalus et al. (2000) ▶ designed a randomized, placebo-controlled trial to evaluate the effect of the onion-olive-oil maceration product on arterial blood pressure and blood fluidity in 30 healthy subjects; in this study, the intervention was done within 5 hr. Results of this study showed a significant reduction in systolic blood pressure and plasma viscosity. There were no significant differences in the diastolic blood pressure, haematocrit and erythrocyte aggregation between two groups (Kalus et al., 2000 ▶).

In our study, similar to the study of Nozawa, blood viscosity significantly decreased in the intervention group. It should be noted that Nozawa’s study was conducted on healthy human and other factors affecting blood fluidity were not measured. In the study done by Kalus et al., participants were healthy subjects and the duration of the intervention was very short. If the participants continued to receive the onion-olive-oil maceration capsule, haematocrit and erythrocyte aggregation could also decrease.

In our study, compared with previous studies, the sample size was greater, the intervention period was longer, and more related variables were investigated. Also, our study participants were diagnosed with polycythemia and both groups performed phlebotomy at the beginning of the study. It has been shown that phlebotomy has a marked influence on the rheological properties of blood by significantly lowering WBV, plasma viscosity, Hct and fibrinogen (Holsworth et al., 2014 ▶). In addition, decreasing iron storage due to phlebotomy significantly modify the concentration of triglycerides without significantly modifying the total cholesterol (Assi and Baz, 2014). The results of our study showed a significant reduction in WBV, Hct, fibrinogen, and TG in both groups, indicating the role of phlebotomy in reducing these rheological parameters.

A plant-based food contains considerable amounts of fiber, minerals, antioxidant vitamins and phytochemicals (Slavin and Lloyd, 2012 ▶). Recent research has shown that the raw ingredients of vegetable soup exert potentially valuable effects. Barley, celery, and lettuce have antioxidant vitamins such as vitamin C (ascorbic acid), and vitamin E (tocopherols and tocotrienols), and decreased serum total cholesterol, triglycerides, LDL and VLDL and increased HDL level (Nicolle et al., 2004 ▶; Březinová Belcredi et al., 2010 ▶; Al-Snafi, 2015 ▶). Onion has antioxidant, antithrombotic, antibacterial, anti-cholesterol, anti-diabetes, anti-proliferative, anti-hypertensive, and anti-obesity properties; also, it inhibits platelet aggregation and improves blood fluidity (Kalus et al., 2000 ▶; Kim and Yim, 2015 ▶). The active compound of carrot is potassium, vitamins such as vitamins A and C, pectin, and antioxidants like beta-carotene. Carrot improves the function of endothelial cells, helps blood vessels to dilate and regulates fluid balance (Kaur and Khanna, 2012 ▶). Parsley, coriander, and dill also have antioxidant activity, fibers and phytoconstituents like β-carotene, apiol, vitamin C, and vitamin E, polyunsaturated fatty acids (PUFA), and exert hypolipidemic and hypoglycemic effects (Singh et al., 2005 ▶; Bahnas et al., 2009 ▶; Laribi et al., 2015 ▶). 

Olive oil and Citrus lemon are strong antioxidants due to their phenolic compounds; also, they improve the lipoprotein profile, glucose metabolism, blood pressure and antithrombotic profile (Moreno-Luna et al., 2012 ▶; Rafiq et al., 2016 ▶). Cinnamon, turmeric, and black pepper have antioxidant, anti-hyperlipidemic, and anti-inflammatory properties. Cinnamon and black pepper also reduce glucose levels (Gullapalli et al., 2013 ▶; Noorafshan and Ashkani-Esfahani; 2013 ▶; Gorgani et al., 2017 ▶). 

The phytochemical components of plants have broad biological properties. They increase the activity of vitamin C, act as antioxidants, prevent LDL cholesterol oxidation to unsafe cholesterol oxides, reduce the production of cholesterol, provide protection against oxidative damage, stimulate immune function, and also have anti-inflammatory and antitumor properties (Cote, 2013 ▶). Several mechanisms are involved in blood rheology impairment via oxidative stress, such as platelet aggregation and elevation of plasma viscosity (Hitsumoto, 2017 ▶). Oxidative stress is defined as an imbalance between the generation of free radicals and reactive metabolites that are often named as oxidants while antioxidants act as a defense system for destruction of these metabolites (Kim and Yim, 2015). Oxidative stress damages important biomolecules such as DNA, lipids, and proteins. While scavenging free radical, erythrocytes become damaged by oxidation, which expends endogenous substances with reducing properties and reduces the level of erythrocyte antioxidant glutathione and superoxide dismutase. The depletion of the antioxidant is accompanied by changes in hemorheological parameters (Gyawali and Richards, 2015 ▶). 

There is a positive correlation between total cholesterol and TG concentrations, and plasma or blood viscosity. High levels of TC and LDL-C impair permeability of erythrocytes and induce erythrocyte aggregation (Seki et al., 2006 ▶). Furthermore, high levels of Hct accelerate atherogenesis, by increasing serum lipids and deposition of large plasma proteins and platelets on the endothelium (Skretteberg et al., 2010 ▶). Following consumption of green vegetables, decreases in saturated fatty acids (SFAs) and increases in PUFAs found in erythrocyte membrane phospholipids, may be associated with rheological and functional changes in red blood cells in hypercholesterolemic patients (Okita et al., 2000 ▶). Lipid peroxidation of polyunsaturated lipids is one of the oxidative stress markers. The end-products of lipid peroxidation, including malondialdehyde (MDA), 4-hydroxy-2-nonenol (4-HNE), and F2-isoprostanes, are detectable in blood and have been used as a measure of oxidative stress (Rahal et al., 2014 ▶). MDA leads to protein crosslinking, and changes the membrane structure of erythrocytes and affects transmembrane transport, consequently has a negative effect on membrane viscoelasticity, surface area to cell volume ratio, and inner viscosity (Kasperczyk et al., 2014 ▶). Peripheral blood circulation facilitates the exchange of oxygen and nutrients between tissue and blood. Oxidative damage to the erythrocyte membrane impairs the flexibility of normal erythrocyte membrane (Nozawa et al., 2007 ▶). 

As noted, all the ingredients of our examined diet have antioxidant and lipid-lowering properties which can explain the positive effect of this functional food on hemorheological parameters.

This study discovered the positive effect of a functional food on blood rheology. Similar studies have not been conducted to assess food product effects in patients with polycythemia and this study is the first clinical trial in this field. The implications of this study can be beneficial for physicians and patients to pay attention to the important role of diet in maintaining health. This study will help the researchers to uncover the critical area of prevention and treatment of diseases associated with blood viscosity; however, more research are required to be done in this regard in the future. 
